# *Casuarinicola australis* Taylor, 2010 (Hemiptera: Triozidae), newly recorded from New Zealand

**DOI:** 10.3897/BDJ.1.e953

**Published:** 2013-09-16

**Authors:** Stephen E. Thorpe

**Affiliations:** † School of Biological Sciences (Tamaki Campus), University of Auckland, Auckland, New Zealand

**Keywords:** *Casuarinicola australis*, *Casuarina*, New Zealand, Auckland, new record, Triozidae

## Abstract

The presence in New Zealand of the triozid *Casuarinicola australis* Taylor, 2010 is reported for the first time, based on new material from Auckland. This is also the first record of the genus from New Zealand.

## Introduction

*Casuarinicola australis* Taylor, 2010 was described from Australia, where it is the most common and widespread member of its genus, being widely distributed in New South Wales, Queensland, South Australia, Victoria and Western Australia. Like its congeners, it is restricted to host trees of the genus *Casuarina*. *Casuarinicola australis* occurs on all Australian species of *Casuarina*, including *Casuarina cunninghamiana* and *Casuarina glauca*. Both these species of *Casuarina* are exotic and present in the wild in New Zealand, according to the New Zealand Organisms Register (NZOR), as well as present in cultivation. *Casuarinicola* has not been reported (by name) previously from N.Z.

## Taxon treatments

### Casuarinicola australis

Taylor, 2010

#### Materials

- **Type status:** Other material. **Occurrence:** recordedBy: Stephen Thorpe; sex: 1 male, 1 female; **Location:** country: New Zealand; verbatimLocality: Mechanics Bay, Auckland City; verbatimElevation: 0-5 m; verbatimLatitude: 36.8474938105S; verbatimLongitude: 174.7869624545E; **Event:** eventDate: 6 January 2013; **Record Level:** institutionCode: Auckland Museum
- **Type status:** Other material. **Occurrence:** recordedBy: Stephen Thorpe; individualCount: 1; sex: female; **Location:** country: New Zealand; verbatimLocality: Felton Mathew Avenue, Saint Johns, Auckland; verbatimLatitude: 36.8741794382S; verbatimLongitude: 174.8506522179E; **Event:** eventDate: 2013-02-20; **Record Level:** institutionCode: Auckland Museum
- **Type status:** Other material. **Occurrence:** recordedBy: Stephen Thorpe; individualCount: many; sex: males, females; **Location:** country: New Zealand; verbatimLocality: Thomas Bloodworth Park, Auckland; verbatimElevation: 0-5 m; verbatimLatitude: 36.8652411423S; verbatimLongitude: 174.7900235653E; **Event:** eventDate: 2013-02-26; **Record Level:** institutionCode: Auckland Museum

#### Description

**Figure 1.**
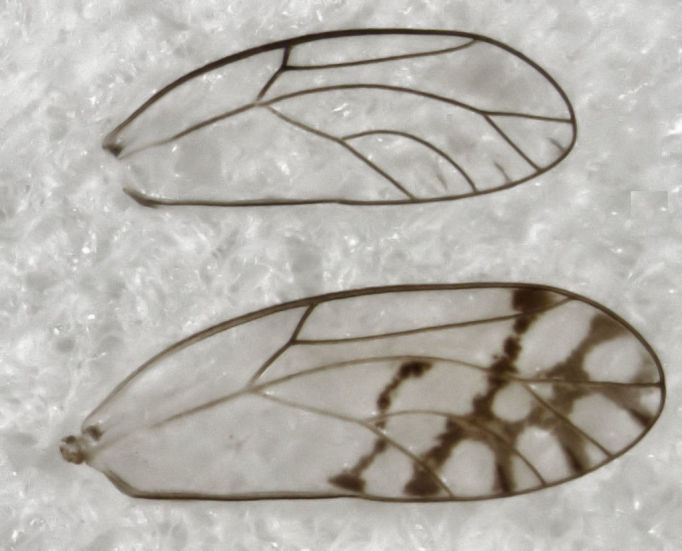
*Casuarinicola australis*, fore wing of male (above) and female (below, length about 2.4 mm).

**Figure 2.**
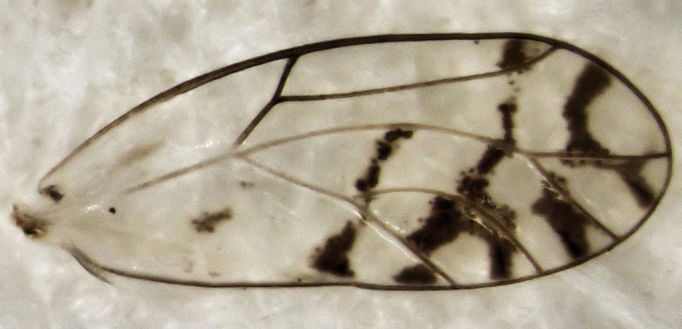
*Casuarinicola australis*, fore wing of female from Saint Johns, 20 Feb 2013.

On 6 Jan 2013, I examined some *Casuarina glauca* trees growing in the vicinity of Ports of Auckland at Mechanics Bay. A few psylloids were observed, including a pair *in copula*, which I collected. The specimens will be vouchered in Auckland Museum. They are easily identified as *Casuarinicola australis* Taylor in Taylor et al. (2010). According to the original description, "this species can be distinguished from all other species in the genus by the female having three incomplete black terminal bands in the fore wing. The male has clear wings." Actually, the bands on the female fore wing are brown, not "black", and the male fore wing is not completely "clear", having three streaks between the veins, as is obvious from the accompanying figures therein (figs. 3-6). Nevertheless, my material (see Fig. 1) exactly matches figs. 5 and 6 in Taylor et al. (2010), and certainly keys out to *Casuarinicola australis* therein, except only that the second and third bands on the female fore wings of my specimen are narrowly joined. Such minor variation is entirely expected for insect colour patterns, and the pattern is still by far closest to that of typical *Casuarinicola australis* than it is to any other species of *Casuarinicola*. I can find no other differences. I therefore recommend that *Casuarinicola australis* be added to the New Zealand Organisms Register (NZOR) as present in the wild. Its "origin" is "exotic". Subsequently, on 20 Feb 2013, a further female specimen was found on a *Casuarina* tree in the Auckland suburb of Saint Johns. The second and third bands on the fore wings are separated, though narrowly (Fig. 2). On 26 February 2013, the species was found to be fairly common on *Casuarina* trees at Thomas Bloodworth Park, Auckland. Several pairs were observed *in copula*. One female was collected. It has the second and third bands of one forewing joined, and of the other narrowly separated. On *Casuarina* in Auckland, there is also an apparently undescribed Australian species of *Trioza*, which is more common and widespread than *Casuarinicola australis*.

## Supplementary Material

XML Treatment for Casuarinicola australis
